# Mortality risk factors of COVID-19 infection in kidney transplantation recipients: a systematic review and meta-analysis of cohorts and clinical registries

**DOI:** 10.1038/s41598-021-99713-y

**Published:** 2021-10-08

**Authors:** Suwasin Udomkarnjananun, Stephen J. Kerr, Natavudh Townamchai, Paweena Susantitaphong, Wasee Tulvatana, Kearkiat Praditpornsilpa, Somchai Eiam-Ong, Yingyos Avihingsanon

**Affiliations:** 1 Division of Nephrology, Department of Medicine, Faculty of Medicine, Chulalongkorn University and King Chulalongkorn Memorial Hospital, 1873 Rama IV Road, Bangkok, 10330 Thailand; 2 Renal Immunology and Transplantation Research Unit, Faculty of Medicine, Chulalongkorn University, Bangkok, Thailand; 3 Excellence Center for Solid Organ Transplantation, King Chulalongkorn Memorial Hospital, Bangkok, Thailand; 4 Research Affairs, Faculty of Medicine, Chulalongkorn University, Bangkok, Thailand; 5 The Kirby Institute, University of New South Wales, Sydney, NSW Australia; 6 Department of Ophthalmology, Faculty of Medicine, Chulalongkorn University and King Chulalongkorn Memorial Hospital, Bangkok, Thailand

**Keywords:** Renal replacement therapy, Risk factors, SARS-CoV-2

## Abstract

Kidney transplantation recipients (KTR) with coronavirus disease 2019 (COVID-19) are at higher risk of death than general population. However, mortality risk factors in KTR are still not clearly identified. Our objective was to systematically analyze published evidence for risk factors associated with mortality in COVID-19 KTR. Electronic databases were searched for eligible studies on 1 August 2021. All prospective and retrospective studies of COVID-19 in KTR were considered eligible without language restriction. Since data in case reports and series could potentially be subsets of larger studies, only studies with ≥ 50 patients were included. Random-effects model meta-analysis was used to calculate weighted mean difference (WMD) and pooled odds ratio (OR) of factors associated with mortality. From a total 1,137 articles retrieved, 13 were included in the systematic review and meta-analysis comprising 4,440 KTR. Compared with survivors, non-survivors were significantly older (WMD 10.5 years, 95% CI 9.3–11.8). KTR of deceased donor were at higher risk of death (OR 1.73, 95% CI 1.10–2.74). Comorbidities including diabetes mellitus, cardiovascular disease, and active cancer significantly increased mortality risk. KTR with dyspnea (OR 5.68, 95% CI 2.11–15.33) and pneumonia (OR 10.64, 95% CI 3.37–33.55) at presentation were at higher mortality risk, while diarrhea decreased the risk (OR 0.61, 95% CI 0.47–0.78). Acute kidney injury was associated with mortality (OR 3.24, 95% CI 1.36–7.70). Inflammatory markers were significantly higher in the non-survivors, including C-reactive protein, procalcitonin, and interleukine-6. A number of COVID-19 mortality risk factors were identified from KTR patient characteristics, presenting symptoms, and laboratory investigations. KTR with these risk factors should receive more intensive monitoring and early therapeutic interventions to optimize health outcomes.

## Introduction

Coronavirus disease 2019 (COVID-19) is an ongoing global pandemic caused by severe acute respiratory syndrome coronavirus-2 (SARS-CoV-2). Elderly patients and patients with multiple comorbidities are known to be at higher risk of death^1, 2^. Immunocompromised patients, particularly solid organ transplantation recipients and those with malignancies, are also at increased risk of severe COVID-19 disease and death^3^.

The mortality rate of COVID-19 In kidney transplantation patients was 20–40%^4–7^, compared with 10–15% mortality rate amongst admitted patients overall^8–10^. The immunosuppressed status in kidney transplantation recipients (KTR) might contribute to the higher mortality rate. Empirical stepwise reductions in immunosuppressive therapy have been recommended in patients at high risk of developing severe disease, and as clinical severity of COVID-19 symptoms increases^11, 12^. However, temporary reductions in immunosuppression might place these patients at risk for allograft rejection thereafter. More data from clinical studies are urgently needed regarding the management of COVID-19 in KTR, including patient selection criteria for immunosuppressive lowering strategies. Patients with higher mortality risk should be treated more aggressively compared to patients with a lower risk. Moreover, many transplantation programs have been halted during the COVID-19 pandemic. Apart from the strain on hospital facilities by general COVID-19 patients, concerns have been raised regarding donor-derived COVID-19 infection in recipients who might need relatively intensified immunosuppression in the perioperative period, although there are currently no reports of such cases^13–15^. Induction therapy might also increase the risk of acquiring COVID-19 in the early post-transplantation period^16^. A clinical tool that identifies patients who are more likely to have a good prognosis of COVID-19 with minimal clinical symptoms after transplantation, might help transplant programs to continue performing kidney transplantation in these low risk patients.

To date, many case reports, case series, and cohort studies of COVID-19 in KTR have been published. However, the clinical risk factors for mortality in KTR with COVID-19 infection are still unclear due to many scattered case reports, and inconsistent reporting with varying quality across larger studies. The objective of this systematic review and meta-analysis was to clarify risk factors for mortality in KTR with COVID-19 infection, to improve of quality of care during the ongoing COVID-19 pandemic.

## Methods

### Data sources and searches

This systematic review was conducted according to the Preferred Reporting Items for Systematic Reviews and Meta-Analysis (PRISMA)^17^. MEDLINE, Scopus, and Cochrane Central Register of Controlled Trials electronic databases were searched for eligible studies on 1 August 2021. The following search strategy was used for MEDLINE: ("Kidney Transplantation"[Mesh]) AND ("COVID-19"[Mesh] OR "SARS-CoV-2"[Mesh]), and the search terms in Scopus were (TITLE-ABS-KEY (COVID-19) AND TITLE-ABS-KEY (kidney AND transplantation)). The MeSH descriptors which exploded all trees of [Coronavirus] and [Kidney Transplantation] were applied in the Cochrane Central register of Controlled Trials. The reference lists in the qualified articles were also reviewed and studies were manually added if deemed appropriate.

### Study selection

This systematic review and meta-analysis focused on risk factors contributing to mortality in COVID-19 KTR. Our inclusion criteria were studies of COVID-19 in KTR that included ≥ 50 patients, that reported numbers of survivors and non-survivors, and demographic and/or clinical characteristics by survival group. Baseline demographic characteristics, clinical presentation, laboratory investigations, and treatments related to transplantation and COVID-19 care were extracted separately for survivors and non-survivors. As single case reports and small case series could be subsets of larger clinical registries, we selected only studies with ≥ 50 KTR patients for our review. In addition, meta-analysis of studies that include studies with a small sample size are at risk for bias caused by sampling error and random variation^18^. Studies with ≥ 50 patients were excluded if they were subsets of other larger studies, based on the study site, start and end date, or if clearly mentioned in the larger studies. If a potentially duplicated population was presented in 2 large studies, the study reporting more information regarding survivors and non-survivors was selected as the main data source. Only studies with adequate information, in accordance with the Strengthening the Reporting of Observational Studies in Epidemiology (STROBE) statement, were included in the review^19^. Two authors (S.U. and S.K.) independently screened the titles and abstracts of the electronic citations, and full-text articles were retrieved for comprehensive review. Disagreements were resolved through consensus and arbitration by a third author (N.T.).

### Data extraction and quality assessment

The following information was extracted from each study: author names, publication date, journal title, study site, country of origin, study duration, total KTR included, and the number of COVID-19 survivors and non-survivors. Baseline patient characteristics, clinical presentations, laboratory investigations, and treatments were retrieved separately for each study, and grouped by survival group. The Newcastle–Ottawa scale was used for the quality assessment of each individual included study^20, 21^. The tool evaluates 3 domains which are selection, comparability, and outcome. Each domain is rated total scores of 4 in the selection domain, 2 in the comparability domain, and 3 in the outcome domain.

### Data synthesis and analysis

Using data reported in each individual study, we used random-effects models to calculate pooled weighted mean differences (WMD) of continuous variables, and pooled odds ratio (OR) for binary variables, for non-survivors versus survivors. Mean and standard deviations (SD) were estimated by the method of Wan et al.^22^, if only the median and range, or interquartile range were provided in the study. Pooled OR were calculated using the logarithm of effect size and standard error from each study. Heterogeneity of pooled effect sizes was assessed using the *I*^2^ index and the Q-test p-value. An *I*^2^ index higher than 75% indicates medium to high heterogeneity. Even when heterogeneity was low or absent, we reported random effects over fixed effects models because clinical care may have differed by different sites, and care practices likely changed as physicians gained more experience in caring for COVID-19 patients. Regression-based Egger’s test was used to test for small-study effects. The mortality change rate over the study period was calculated by regressing the proportion of deaths against the study end dates, using a generalized linear model with a binomial family and logit link functions, and robust variance estimates, after weighting by study size^23^. The analyses were performed using Stata Statistical Software Release 16.1 (StataCorp LLC, College Station, TX).

### Ethical considerations

This meta-analysis and systematic review did not directly obtain data from human or animal subject. All of the included studies’ information was published in the scientific journals without the possibility to identify the individual patients. The clinical and research activities being reported are consistent with the Principles of the Declaration of Istanbul as outlined in the 'Declaration of Istanbul on Organ Trafficking and Transplant Tourism'.

## Results

### Characteristics of the included studies

**Figure 1:**
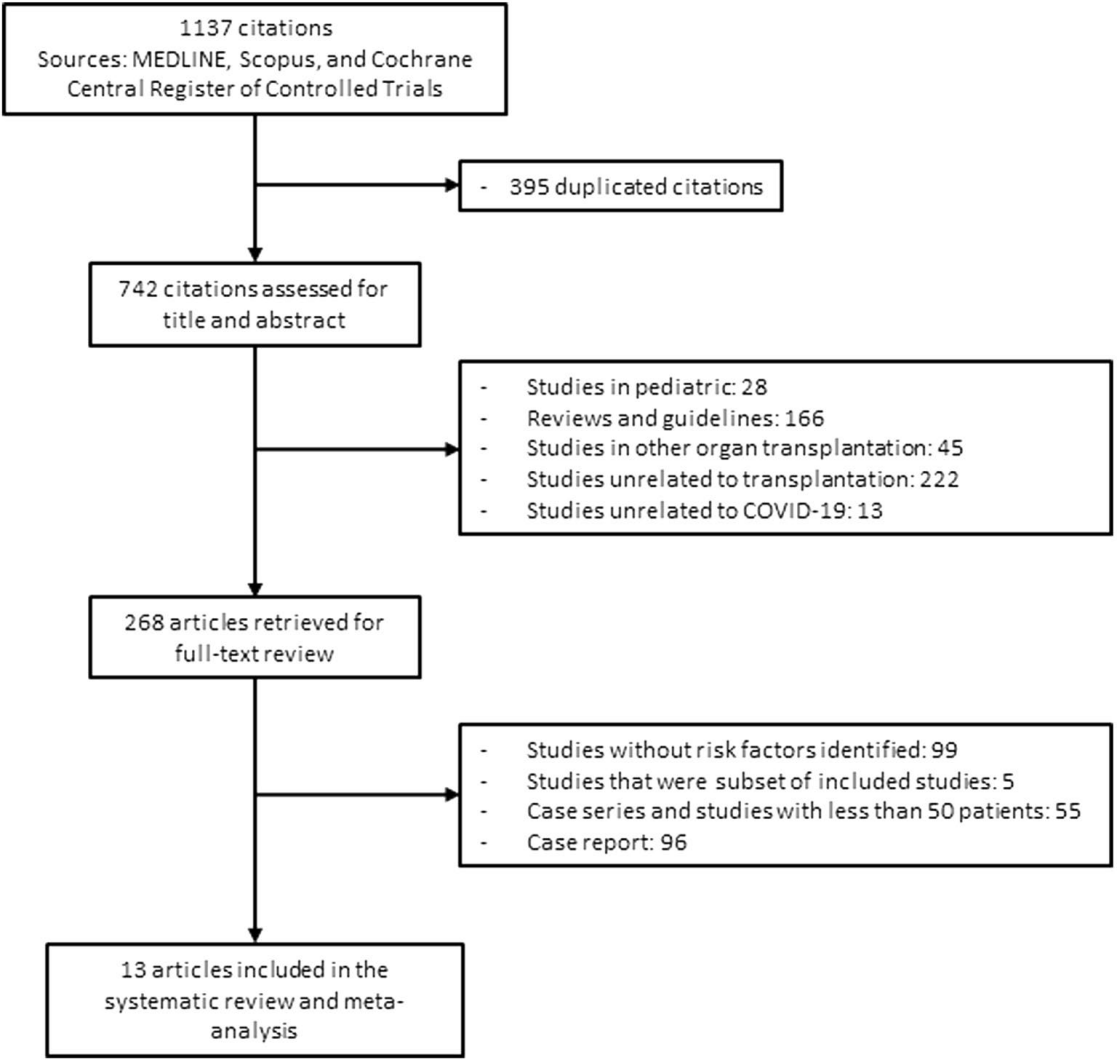
Flow diagram of study selection.

Figure 1 shows the flow diagram of study selection. A total 1,137 studies were retrieved using our search criteria. After duplicate citations and irrelevant studies were excluded, 268 articles underwent full-text review, and 13 articles were included in the final meta-analysis^24–36^. Details of each study are displayed in Table 1. Most studies were conducted during the first wave of the COVID-19 pandemic: the last study end date was December 2020, and study duration ranged from 1 to 9 months. Ten of 13 studies were multicenter, including 1 multi-continent international study and 1 multi-country European study. KTR numbers included in each study ranged from 52 to 1,680 patients, and mortality rates ranged from 12 to 32%. The total number of KTR reported in our meta-analysis was 4,440. The Newcastle–Ottawa Quality Assessment Scale of the included studies are shown in the Supplementary Table S1.

**Table 1:** Summary characteristics of included studies.

References	Authors	Journal	First published	Study center	Country of cohort	Study duration	Total kidney transplantation patients included	Survivors	Non-survivors
^24^	Bossini et al	American Journal of Transplantation	July 2020	Multicenter	Italy	1/3/20 to 16/4/20	53	38 (72%)	15 (28%)
^25^	Cravedi et al	American Journal of Transplantation	July 2020	Multicenter	International	2/3/20 to 15/5/20	144	98 (68%)	46 (32%)
^26^	Caillard et al	Kidney International	August 2020	Multicenter	France	1/3/20 to 21/4/20	243	200 (82%)	43 (18%)
^27^	Azzi et al	Kidney International	October 2020	Albert Einstein College of Medicine, New York	USA	16/3/20 to 29/7/20	229	182 (79%)	47 (21%)
^28^	Craig-Schapiro et al	American Journal of Transplantation	October 2020	Weill Cornell Medicine, New York	USA	13/3/20 to 20/5/20	52	39 (75%)	13 (25%)
^29^	Hilbrands et al	Nephrology Dialysis Transplantation	October 2020	Multicenter	Europe	1/2/20 to 1/6/20	305	240 (79%)	65 (21%)
^30^	Mamode et al	Transplantation	November 2020	Multicenter	UK	1/3/20 to 27/4/20	121	85 (70%)	36 (30%)
^31^	Willicombe et al	Transplantation	November 2020	Imperial College, London	UK	1/6/20 to 3/7/20	113	96 (85%)	17 (15%)
^32^	Oto et al	BMC Nephrology	March 2021	Multicenter	Turkey	17/4/20 to 1/6/20	109	95 (87%)	14 (13%)
^33^	Kute et al	Transplantation	April 2021	Multicenter	India	23/3/20 to 15/9/20	250	221 (88%)	29 (12%)
^34^	Villanego et al	American Journal of Transplantation	April 2021	Multicenter	Spain	18/3/20 to 5/12/20	1,011	791 (78%)	220 (22%)
^35^	Alshaqaq et al	Annals of Transplantation	June 2021	Multicenter	Saudi Arabia	1/3/20 to 31/8/20	130	116 (89%)	14 (11%)
^36^	Requiao-Moura et al	PLoS ONE	July 2021	Multicenter	Brazil	1/3/20 to 11/11/20	1,680	90-day cumulative incidence of death 21%	

### Meta-analysis of patient characteristics associated with mortality of KTR with COVID-19 infection

Pooled estimates of patient characteristics associated with morality, with the numbers of studies and patients reporting data are displayed in Table 2. The median number of studies reporting these clinical characteristics was 6 (range 3–10), and median number of patients was 1,514 (range 496–2577). Non-survivors were significantly older than survivors, with WMD of 10.5 years (95% CI 9.3–11.8; p-value < 0.001; *I*^2^ 0%; Q-test p-value = 0.46). KTR with co-existing medical comorbidities were at higher risk for death, including diabetes mellitus (OR 1.80; 95% CI 1.43–2.26; p-value < 0.001; *I*^2^ 0%; Q-test p-value 0.58), cardiovascular diseases (OR 2.21; 95% CI 1.60–3.06; p-value < 0.001; *I*^2^ 15.3%; Q-test p-value 0.35), and active cancer (OR 2.00; 95% CI 1.05–3.80; p-value = 0.034; *I*^2^ 41.2%; Q-test p-value 0.11). Patients who received kidney allografts from deceased donors had 1.73-fold higher odds of mortality (95% CI 1.10–2.74; p-value = 0.019; *I*^2^ 5%; Q-test p-value 0.40).

**Table 2:** Meta-analysis of patient characteristics between survivor and non-survivor kidney transplantation patients.

Variables	Survivors	Non-survivors	Number of patients reported	Number of studies reporting	Weighted mean difference (95% CI)	p-value from random effects model	*I*^2^ index (%)	Q-test p-value	Egger’s test p-value
Age, years (mean ± SD)	54.9 ± 15.4	67.5 ± 11.8	2,577	10	10.5 (9.3, 11.8)	** < 0.001**	0	0.46	0.04
BMI, kg/m^2^ (mean ± SD)	27.5 ± 5.2	27.8 ± 6.0	586	3	− 0.1 (− 0.3, 0.2)	0.46	0	0.75	0.52
Post kidney transplantation duration, months (mean ± SD)	76.5 ± 78.0	79.4 ± 91.0	1,909	7	3.6 (− 4.9, 12.2)	0.40	0	0.79	0.87
Onset to admission, days (mean ± SD)	8.0 ± 6.4	4.6 ± 3.4	496	3	− 3.7 (− 8.2, 0.91)	0.12	94.2	0.01	< 0.001

Variables	Survivors	Non-survivors	Number of patients reported	Number of studies reporting	Pooled odds ratio (95% CI)	p-value from random effects model	*I*^2^ index (%)	Q-test p-value	Egger’s test p-value
Male (%)	1,345 (65.7)	341 (64.3)	2,577	10	0.94 (0.75, 1.19)	0.63	10.6	0.37	0.38
Hypertension (%)	928 (76.3)	243 (81.8)	1,514	8	0.99 (0.68, 1.43)	0.94	0	0.95	0.93
Diabetes mellitus (%)	548 (26.8)	204 (38.5)	2,577	10	1.80 (1.43, 2.26)	** < 0.001**	1.5	0.58	0.08
Obesity (%)	395 (46.8)	106 (49.8)	1,063	5	1.30 (0.92, 1.82)	0.14	0	0.24	0.15
Cardiovascular diseases (%)	248 (21.2)	106 (38.7)	1,445	8	2.21 (1.60, 3.06)	** < 0.001**	15.3	0.35	0.58
Pulmonary diseases (%)	86 (10.1)	31 (13.6)	1,143	6	1.35 (0.86, 2.13)	0.19	0	0.49	0.53
Active cancer (%)	75 (8.2)	35 (15.1)	1,143	6	2.00 (1.05, 3.80)	**0.034**	41.2	0.11	0.88
History of smoking (%)	262 (30.7)	70 (30.7)	1,082	6	0.96 (0.69, 1.34)	0.82	0	0.74	0.60
Deceased donor kidney transplant (%)	369 (47.5)	130 (68.8)	966	6	1.73 (1.10, 2.74)	**0.019**	5.0	0.40	0.35
Less than 12 months since kidney transplant (%)	220 (11.4)	64 (13.3)	2,404	8	1.27 (0.83, 1.95)	0.28	33.1	0.11	0.004
Lymphocyte depleting induction (%)	432 (68.1)	87 (56.1)	789	6	0.73 (0.49, 1.08)	0.12	0	0.90	0.44
Tacrolimus (%)	1,108 (83.9)	288 (79.6)	1,682	5	0.74 (0.50, 1.10)	0.13	15.8	0.47	0.72
mTORi (%)	284 (16.4)	55 (12.1)	2,183	7	0.89 (0.61, 1.30)	0.55	11.5	0.020	0.09
MPA (%)	1,305 (75.0)	320 (73.7)	2,175	7	0.98 (0.76, 1.25)	0.86	0	0.79	0.99
Prednisolone (%)	1,389 (78.0)	355 (79.4)	2,227	8	1.13 (0.86, 1.48)	0.38	0	0.37	0.84
Fever (%)	1,217 (74.0)	324 (77.7)	2,062	6	1.19 (0.83, 1.69)	0.34	32.1	0.27	0.23
Cough (%)	1,011 (65.4)	274 (73.9)	1,918	5	1.21 (0.73, 2.01)	0.46	65.3	0.017	0.07
Dyspnea (%)	298 (34.9)	146 (74.1)	1,051	5	5.68 (2.11, 15.33)	** < 0.001**	84.6	< 0.001	0.19
Diarrhea (%)	540 (32.8)	100 (24.0)	2,062	6	0.61 (0.47, 0.78)	** < 0.001**	0	0.75	0.31
Pneumonia (%)	582 (55.4)	240 (93.8)	1,306	3	10.64 (3.37, 33.56)	** < 0.001**	25.6	0.18	0.87
Acute kidney injury (%)	203 (40.2)	82 (63.1)	634	5	3.24 (1.36, 7.70)	**0.008**	63.2	0.030	0.002

*BMI* body mass index, *CNI* calcineurin inhibitor, *MPA* mycophenolic acid, *mTORi* mammalian target of rapamycin inhibitor, *SpO*_*2*_ oxygen saturation.

For presenting COVID-19 symptoms, patients with dyspnea and pneumonia were at 5.68-fold (95% CI 2.11–15.33; p-value < 0.001; *I*^2^ 84.6%; Q-test p-value < 0.001) and 10.64-fold (95% CI 3.37–33.56; p-value < 0.001; *I*^2^ 25.6%; Q-test p-value 0.18) higher risk of death, respectively. Acute kidney injury also significantly increased mortality risk (OR 3.24; 95% CI 1.36–7.70; p-value = 0.008; *I*^2^ 63.2%; Q-test p-value 0.030). However, diarrhea significantly lowered the risk with OR of 0.61 (95% CI 0.47–0.78; p-value < 0.001; *I*^2^ 0%; Q-test p-value 0.75). Supplementary Figure S1 shows the forest plot of patient characteristics that were significantly associated with mortality.

### Meta-analysis of baseline laboratory investigations associated with mortality of KTR with COVID-19 infection

Table 3 shows the baseline laboratory results of survivors and non-survivors. Non-survivors had significantly lower estimate glomerular filtration rate (eGFR) compared with the survivors (WMD − 11.4 mL/min/1.73 m^2^; 95% CI − 15.7, − 7.0; p-value < 0.001; *I*^2^ 0%; Q-test p-value = 0.55). Other standard laboratory investigations did not show the association with COVID-19 mortality.

**Table 3:** Meta-analysis of baseline laboratory investigations between survivor and non-survivor kidney transplantation recipient.

Variables	Survivors	Non-survivors	Number of patients reported	Number of studies reporting	Weighted mean difference (95% CI)	p-value from random effects model	*I*^2^ index (%)	Q-test p-value	Egger’s test p-value
Initial serum Cr, mg/dL (mean ± SD)	1.79 ± 0.88	2.19 ± 1.28	825	5	0.50 (− 0.02, 1.03)	0.06	90.9	< 0.001	0.85
Initial eGFR, mL/min/1.73 m^2^ (mean ± SD)	44.4 ± 23.3	34.4 ± 21.0	562	3	− 11.4 (− 15.7, − 7.0)	** < 0.001**	0	0.55	0.72
Hemoglobin, g/dL (mean ± SD)	11.6 ± 2.1	11.4 ± 2.2	582	4	− 0.3 (− 0.9, 0.2)	0.22	34.0	0.20	0.09
Platelet, × 10^3^/μL (mean ± SD)	199 ± 66	172 ± 115	825	5	− 18.5 (− 39.8, 2.8)	0.09	60.1	0.030	0.18
WBC, × 10^3^/μL (mean ± SD)	6.42 ± 2.76	7.90 ± 3.80	525	4	1.61 (− 0.14, 3.36)	0.07	88.0	< 0.001	0.67
Lymphocytes, × 10^3^/μL (mean ± SD)	1.04 ± 1.03	0.77 ± 0.50	1,182	7	− 0.06 (− 0.15, 0.02)	0.16	34.5	0.02	0.004
Lactate dehydrogenase, U/L (mean ± SD)	304.0 ± 85.3	425.1 ± 205.3	473	3	117.4 (− 13.2, 248.0)	0.078	96.1	< 0.001	0.94
C-reactive protein, mg/dL (mean ± SD)	5.66 ± 8.23	10.50 ± 9.42	830	5	4.85 (1.18, 8.52)	**0.010**	88.9	< 0.001	0.18
D-dimer, μg/mL (mean ± SD)	1.29 ± 1.36	1.67 ± 1.53	525	4	0.37 (− 0.22, 0.96)	0.22	88.6	0.002	0.87
Procalcitonin, ng/mL (mean ± SD)	0.20 ± 0.57	0.97 ± 1.48	525	4	0.60 (0.36, 0.83)	** < 0.001**	64.5	0.031	0.77
Ferritin, ng/mL (mean ± SD)	893 ± 1294	1232 ± 1041	634	5	128.5 (− 276.1, 533.1)	0.53	71.5	0.019	0.001
IL-6, pg/mL (mean ± SD)	31.3 ± 35.1	126.0 ± 178.7	473	3	95.4 (54.0, 136.8)	** < 0.001**	76.1	0.006	0.86

*Cr* creatinine, *eGFR* estimated glomerular filtration rate, *WBC* white blood cell.

Details of biomarkers reflecting tissue damage and inflammation at baseline were reported in 3–5 studies representing 473–830 patients in Table 3. Three biomarkers were significantly higher in non-survivors compared with the survived KTR including C-reactive protein (WMD 4.85 mg/dL; 95% CI 1.18–8.52; p-value = 0.010; *I*^2^ 88.9%; Q-test p-value < 0.001), procalcitonin (WMD 0.60 ng/mL; 95% CI 0.36–0.83; p-value < 0.001; *I*^2^ 64.5%; Q-test p-value = 0.031), and IL-6 (WMD 95.4 pg/mL; 95% CI 54.0–136.8; p-value < 0.001; *I*^2^ 76.1%; Q-test p-value = 0.006). Supplementary Figure S2 displays the forest plot of laboratory variables showing a significant association with mortality.

### Meta-analysis of treatment received associated with mortality of KTR with COVID-19 infection

Table 4 shows details of the treatment received in the survivors and non-survivors. A significantly higher proportion of KTR non-survivors required ventilator support or intubation (OR 56.45; 95% CI 9.67–329.62; p-value < 0.001; *I*^2^ 93.1%; Q-test p-value < 0.001). Significantly higher proportions of non-survivors than survivors were treated with hydroxychloroquine, steroids, antibiotics, tocilizumab, and convalescent plasma. Antiviral drugs including lopinavir, remdesivir, darunavir, and favipiravir were given more frequently to non-survivors (OR 1.99; 95% CI 1.36–2.93; p-value < 0.001; *I*^2^ 15.8%; Q-test p-value = 0.17). The forest plot of each significantly associated variable is illustrated in Supplementary Figure S3.

**Table 4:** Meta-analysis of treatment received between survivor and non-survivor kidney transplantation patients.

Variables	Survivors	Non-survivors	Number of patients reported	Number of studies reporting	Pooled odds ratio (95% CI)	p-value from random effects model	*I*^2^ index (%)	Q-test p-value	Egger’s test p-value
Ventilator support or intubation (%)	68 (5.3)	224 (64.0)	1,645	6	56.45 (9.67, 329.62)	** < 0.001**	93.1	< 0.001	0.007
Withhold CNIs (%)	32 (6.9)	46 (39.3)	582	4	10.07 (0.76, 132.62)	0.08	93.1	< 0.001	< 0.001
Withhold antimetabolites (%)	379 (75.2)	110 (84.6)	634	5	1.66 (0.92, 2.99)	0.09	0	0.39	0.46
Hydroxychloroquine (%)	716 (55.3)	226 (64.6)	1,645	6	1.55 (1.20, 2.00)	** < 0.001**	0	0.38	0.29
Steroid (%)	550 (42.5)	240 (68.6)	1,645	6	4.40 (1.70, 11.38)	**0.002**	81.6	0.002	0.014
Antibiotics (%)	553 (44.0)	169 (50.1)	1,593	5	1.91 (1.06, 3.46)	**0.031**	46.3	0.10	0.013
Antivirals (%)	273 (21.1)	107 (30.6)	1,645	6	1.99 (1.36, 2.93)	** < 0.001**	15.8	0.17	0.15
Tocilizumab (%)	120 (9.3)	90 (25.7)	1,645	6	5.40 (1.54, 18.88)	**0.008**	88.1	< 0.001	0.88
Convalescent plasma (%)	8 (2.6)	15 (21.4)	381	3	8.76 (1.85, 41.5)	**0.006**	56.2	0.10	0.86

CNI; calcineurin inhibitor.

### Multivariable models for mortality in each study

Ten of 13 studies presented adjusted (multivariable) models for factors associated with mortality (Table 5)^24–26, 29, 31–36^. Statistical methods to analyze multivariable model were different between studies including logistic regression and Cox proportional hazard regression. Thus, the combined or pooled effect sizes of each variable was not executed. The most common variables significantly contributing to mortality after adjustment were age in 8 studies, followed by dyspnea or respiratory rate (5 studies) and renal function (5 studies), and the presence of cardiovascular disease in 3 studies.

**Table 5:** Multivariable model for mortality in each study.

Authors [reference]	Study center	Country of cohort	Significant variables from multivariable model for mortality	Adjusted odds or hazard ratio (95% CI)	p-value	Model selection method
Bossini et al^24^	Multicenter	Italy	Age > 60 vs < 60	1.12 (1.03–1.24)	0.01	Stepwise selection after including all statistically significant variables from univariate logistic regression
			Shortness of breath	13.7 (2.7–68.9)	0.004	
Cravedi et al^25^	Multicenter	International	Age	1.07 (1.02–1.14)	0.022	Akaike information criterion and Nagelkerke pseudo *R*^2^ after logistic regression
			Respiratory rate ≥ 20 vs < 20	6.88 (1.63–41.98)	0.017	
			IL-6	1 (1–1.01)	0.04	
			eGFR	0.96 (0.93–0.99)	0.029	
Caillard et al^26^	Multicenter	France	Age > 60 vs < 60	3.81 (1.56–9.31)	0.003	Backward selection after including all statistically significant variables from univariate Cox regression
			Cardiovascular disease	2.04 (1.07–3.90)	0.031	
			Dyspnea on admission	2.35 (1.23–4.49)	0.010	
Hilbrands et al^29^	Multicenter	Europe	Age	1.07 (1.04–1.10)	< 0.001	Backward selection after included all statistically significant variables from univariate Cox regression
			Respiratory rate	1.07 (1.03–1.11)	< 0.001	
			> 25% increased creatinine	1.89 (1.05–3.40)	0.03	
			Prednisolone use	2.88 (1.03–8.03)	0.04	
Willicombe et al^31^	Imperial College, London	UK	Age	1.07 (1.00–1.13)	0.041	Backward selection after included all statistically significant variables from univariate logistic regression
			No diabetes mellitus	0.27 (0.07–0.99)	0.047	
			Living donor transplantation	0.08 (0.01–0.72)	0.024	
			Prednisolone use	5.98 (1.65–21.60)	0.006	
Oto et al^32^	Multicenter	Turkey	Presence of ischemic heart disease	4.129 (1.104–15.442)	0.035	Variables with p-value < 0.05 from univariate logistic regression were adjusted in the multivariable model
			Creatinine at presentation	1.681 (1.083–2.608)	0.021	
Kute et al^33^	Multicenter	India	Baseline creatinine before COVID-19	5.424 (1.294–2.273E7)	0.043	Not reported (Cox regression)
Villanego et al^34^	Multicenter	Spain	Age	1.06 (1.05–1.08)	< 0.001	Variables with p-value < 0.1 from univariate analysis were included in the multivariable Cox model
			Time from transplantation ≤ 6 months	1.64 (1.07–2.50)	0.021	
			Gastrointestinal symptoms	0.66 (0.48–0.90)	0.011	
			Pneumonia	5.04 (2.81–9.05)	< 0.001	
Alshaqaq et al^35^	Multicenter	Saudi Arabia	Age	1.06 (1.013–1.109)	0.012	Statistically significant variables and clinically important variables were included in a multivariate Cox regression model
			Creatinine at presentation	1.002 (1.00–1.004)	0.016	
			Use of azathioprine	6.38 (1.374–29.630)	0.018	
			Acute kidney injury	18.11 (2.244–146.21)	0.007	
Requiao-Moura et al^36^	Multicenter	Brazil	Age	1.054 (1.040–1.067)	< 0.001	Variables with p-value ≤ 0.1 from univariate analysis were included in the multivariable logistic regression model
			Time after transplantation (years)	1.025 (1.002–1.047)	0.030	
			Hypertension	1.566 (1.070–2.293)	0.021	
			Cardiovascular disease	1.517 (1.047–2.198)	0.028	
			CNI-MPA combination	1.197 (1.022–1.401)	0.026	
			Recent high dose of steroids	1.534 (1.063–2.214)	0.022	
			Days of symptom before presentation	0.954 (0.928–0.981)	0.001	
			Dyspnea	3.437 (2.584–4.571)	< 0.001	
			Headache	0.552 (0.371–0.821)	0.003	
			Anosmia	0.563 (0.387–0.821)	0.003	

*ARDS* acute respiratory distress syndrome, *CNI* calcineurin inhibitor, *eGFR* estimated glomerular filtration rate, *IL-6* interleukine-6, *LDH* lactate dehydrogenase, *MPA* mycophenolate.

### Mortality trend from the studies of COVID-19 in kidney transplantation recipients

**Figure 2:**
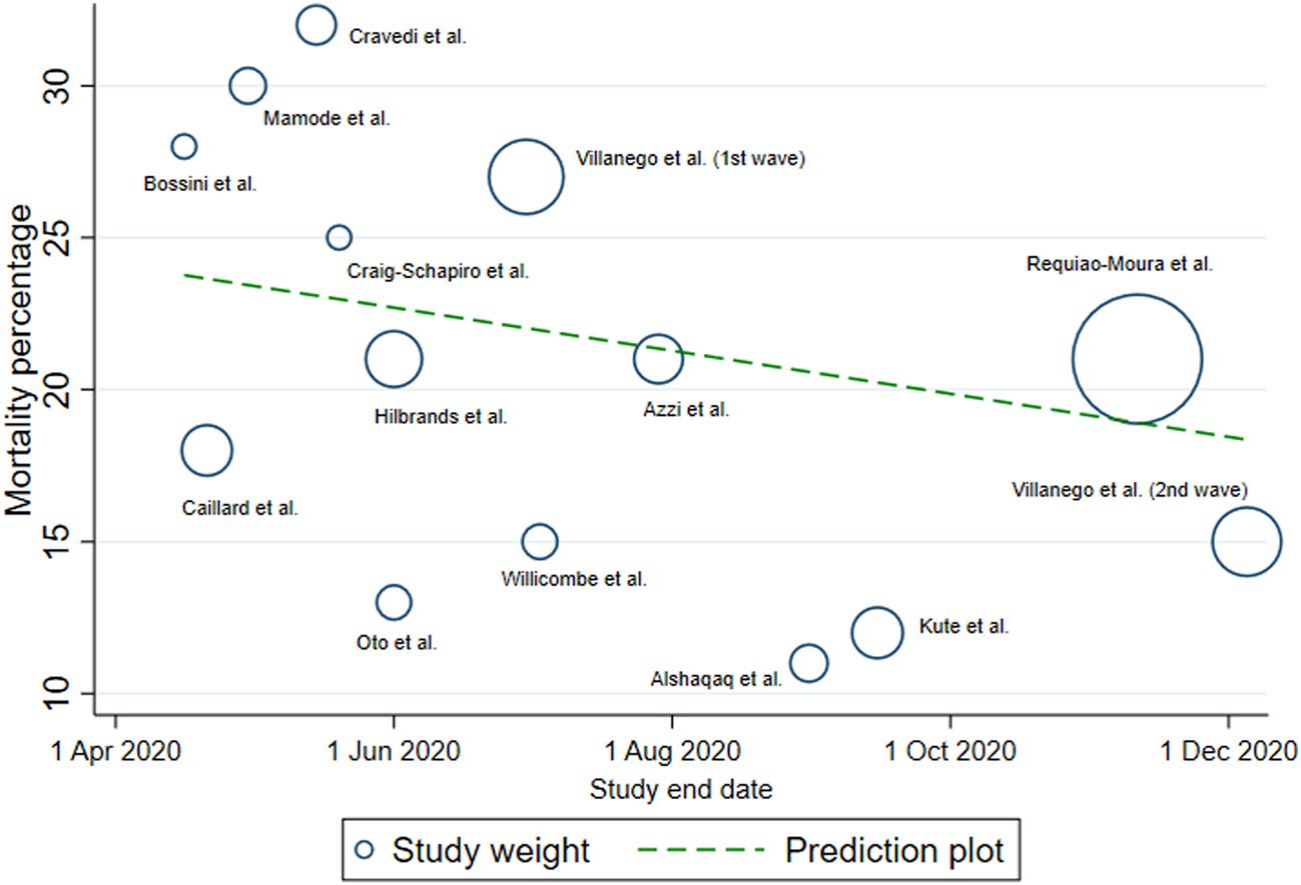
Bubble plot of actual mortality percentage and study end date, with the predicted regression line from a generalized linear model. Bubble size is proportional to the total number of participants in the study.

The mortality percentages from all 13 studies included in this meta-analysis were plotted against the study end date, with the back-transformed predicted slope from the generalized linear model in Fig. 2. A gradual decreasing trend in mortality was noted in the prediction plot. The change in the predicted mortality percentage over the all-study period was -5.1% (regression coefficient per hundred days = − 2.2 (95% CI − 2.4, − 2.0; P < 0.001).

## Discussions

This systematic review and meta-analysis are the first to describe and quantitate the degree of risk for factors associated with mortality in COVID-19 KTR from large cohorts and clinical registries. The results show that a number of baseline patient characteristics increased the risk of patient death, including increasing age, medical comorbidities, and being recipients of deceased donor kidneys. The latter likely relates to inferior allograft function relative to the recipients of living donors^37–41^, which predisposes patients to an increased risk for severe COVID-19. Non-survivors presented with more dyspnea, pneumonia, and acute kidney injury.

Interestingly, gastrointestinal symptoms significantly more frequent in the survivors. This finding is concordant across studies from France, Spain, the European registry, India, and the international cohort^25, 26, 29, 33, 34^. The *I*^2^ index of the gastrointestinal symptoms was 0% indicating very low heterogeneity. Diarrhea in COVID-19 patients is proposed to result from direct infection of COVID-19 in the intestinal epithelium cells, via angiotensin-converting enzyme 2 (ACE2) receptors that further increase local proinflammatory cytokines and changes in the intestinal flora^42, 43^. However, association with lower mortality in patients with diarrhea needs further study to assess whether this association is due to more rapid viral clearance through the gastrointestinal tract, or the stimulation of specific immune cells in the intestinal immune-network.

Obesity is an established risk factor for COVID-19 mortality in the general population^44, 45^. Surprisingly, obesity was not associated with mortality in our meta-analysis of KTR, and the studies reporting on this risk factor had low heterogeneity demonstrated by the *I*^2^ index of 0% in Table 2) ^25, 26, 29, 30, 33^. In addition, no multivariable models in Table 5 included obesity as a significant predictor for mortality. Several mechanisms have been proposed to link obesity to severe COVID-19 illness or death, including lower cardiorespiratory-metabolic reserve and dysfunctional immune response via excess adiposity^46^. It is possible that in KTR, immunosuppression or previous exposure to uremic status, might override or interfere with the effect of ectopic fat deposition that also leads to higher mortality of COVID-19 in KTR compared with the general population^47, 48^. However, further studies are needed to evaluate this hypothesis.

To date, many large randomized controlled trials (RCT) have failed to demonstrate a benefit of treatment interventions in lowering COVID-19 mortality^49–55^. Although there is no evidence that interventions decrease mortality rates, some evidence suggests that treatments including remdesivir, convalescent plasma therapy, and tocilizumab can attenuate the clinical course in COVID-19 patients^56–60^. Dexamethasone is the only medication proven to lower 28-day mortality in COVID-19 patients who received respiratory support^61^. The results from this meta-analysis supports that the non-survivors, compared with survivors, exhibit higher inflammatory states, demonstrated from the significantly higher levels of C-reactive protein, pro-calcitonin, and IL-6. This inflammation cannot be explained from the virus itself, and is thought to result from dysregulation of the host immune response leading to a “cytokine storm” and multiple organ dysfunction^62–64^. The clinical and laboratory results from this study could help identify the COVID-19 KTR with ongoing inflammation who are at risk for multiple organ dysfunction and death, and these patients might be candidates for anti-inflammatory immunomodulator agents. For example, case reports and case series have demonstrated the benefit of tocilizumab as a treatment of COVID-19 KTR^65–68^. More favorable treatment responses might be achieved if IL-6 levels and/or other biomarkers are used as an inclusion criterion for receiving tocilizumab in future RCTs.

It is important not to misinterpret the associations between treatment effects and mortality found in this meta-analysis. None of the included studies were RCTs specifically designed to evaluate treatment outcomes, the pooled univariable OR presented in the results section do not represent causal relationships, since they are confounded by disease severity, with patients experiencing more severe symptoms more likely to receive aggressive treatment interventions. The bubble plot in Fig. 2 reveals possible improvement of the care of COVID-19 KTR, as has been demonstrated in the non-organ transplant COVID-19 patients^8, 69^.

The information from this systematic review and meta-analysis could be used in many ways. Clinician could identify patient characteristics suggesting a poor prognosis, and begin early aggressive monitoring and treatment. According to our results, KTR at higher risk of death included the elderly (mean age of the non-survivors was 67.5 years compared with 54.9 years in the survivor group), patients with diabetes mellitus or cardiovascular diseases, patients with active malignancy, history of deceased donor kidney transplantation, dyspneic patients, presence of pneumonia, and patients with acute kidney injury on initial presentation, or with low eGFR (mean eGFR of the non-survivors was 34.4 mL/min/1.73 m^2^ compared with 44.4 mL/min/1.73 m^2^ in the survivor group). In addition, elevated concentrations of C-reactive protein, procalcitonin, and IL-6 at presentation should flag the patient as having an increased risk of death. In-hospital treatment with more aggressive and earlier immunosuppression reduction strategies would be reasonable in these high-risk patients, who also might be appropriate targets for the future studies of novel therapeutic interventions. Patients without any of these high-risk features could be closely monitored without significant changes in their immunosuppression. This strategy should be tested in future cohorts or clinical trials. Given the slow role out of vaccinations in many countries, patients at high risk of mortality should be prioritized to receive vaccinations. In addition, transplant centers could resume kidney transplantation programs in potential recipients who are at low COVID-19 mortality risk, based on an assessment of risks in baseline patient characteristics, accompanied by proper decision-making systems^70, 71^.

There are some limitations in our study. First, the possibility of duplicated information in individual patient level data could not be completely eliminated across our included studies, particularly international study^25^. However, we carefully selected only large studies with ≥ 50 patients, and thoroughly reviewed all article characteristics to remove studies that were subsets of others, thereby minimizing the chance of data duplication. Using this method, 5 studies were excluded as shown in Fig. 1. No included studies contained identical data and had distinctive information that was valuable for the meta-analysis. Second, SARS-CoV-2 viral load was not routinely measured, so correlations between viral load and KTR outcomes are lacking. Recent studies have demonstrated that the degree of SARS-CoV-2 viral load elevation correlated with disease severity and mortality^72^, therefore the viral load is likely to be another important factor predicting mortality risk in COVID-19 KTR. Third, the included studies were mainly from the European countries and the USA, with only limited data from Asian countries. Fourth, Egger’s test is not reliable for assessing publication bias when small study numbers are involved. However, given the interest of the medical community in reporting studies about COVID-19, we think that the risk of publication bias is mitigated. Lastly, the complications of COVID-19 contributing to patient death, such as superimposed bacterial infection and thromboembolic phenomenon, were not adequately reported in the included studies^73, 74^. More robust reporting of these complications would raise the awareness amongst clinicians regarding these potentially fatal complications.

In conclusion, mortality risk of COVID-19 KTR was increased in older patients, with medical comorbidities and deceased donor kidney recipients. Those with acute kidney injury, dyspnea, pneumonia, and increased of inflammatory biomarkers also had an increased risk of dying. Gastrointestinal tract symptoms were associated with lower risk of death. These risk factors could be used for developing clinical scores to further improve the quality of care in COVID-19 kidney transplant patients.

## Supplementary Information

Supplementary Information.

## Data Availability

The datasets generated during and/or analyzed during the current study are available from the corresponding author on reasonable request.
