# Supplementary material for: Mortality risk factors of COVID-19 infection in kidney transplantation recipients: a systematic review and meta-analysis of cohorts and clinical registries
Source: Sci Rep. 2021 Oct 8;11:20073. doi: 10.1038/s41598-021-99713-y (PMC8501014; doi:10.1038/s41598-021-99713-y)

Mortality Risk Factors of COVID-19 Infection in Kidney Transplantation Recipients: A Systematic Review and Meta-Analysis of Cohorts and Clinical Registries

Suwasin Udomkarnjananun, MD,[1,2,3] Stephen J. Kerr, PhD,[4,5] Natavudh Townamchai, MD,[1,2,3] Paweena Susantitaphong, MD, PhD,[1] Wasee Tulvatana, MD,[6] Kearkiat Praditpornsilpa, MD,[1] Somchai Eiam-Ong, MD,[1] Yingyos Avihingsanon, MD[1,2,3]

[1]Division of Nephrology, Department of Medicine, Faculty of Medicine, Chulalongkorn University and King Chulalongkorn Memorial Hospital, Bangkok, Thailand

[2]Renal Immunology and Transplantation Research Unit, Faculty of Medicine, Chulalongkorn University, Bangkok, Thailand

[3]Excellence Center for Solid Organ Transplantation, King Chulalongkorn Memorial Hospital, Bangkok, Thailand

[4]Research Affairs, Faculty of Medicine, Chulalongkorn University, Bangkok, Thailand

[5]The Kirby Institute, University of New South Wales, Sydney, NSW, Australia

[6]Department of Ophthalmology, Faculty of Medicine, Chulalongkorn University and King Chulalongkorn Memorial Hospital, Bangkok, Thailand

Correspondence: Suwasin Udomkarnjananun, Division of Nephrology, Department of Medicine, Faculty of Medicine, Chulalongkorn University, 1873 Rama IV Road, Bangkok, Thailand,10330.

Supplementary Table S1: Quality assessment of the included studies using the Newcastle-Ottawa Quality Assessment Scale.

**Authors**	**Selection**<br><br>**(From total of ●●●●)**	**Comparability**<br><br>**(From total of ●●)**	**Outcome**<br><br>**(From total of ●●●)**
Bossini et al.	●●●●	●●	●●
Cravedi et al.	●●●●	●●	●●●
Caillard et al.	●●●●	●●	●●●
Azzi et al.	●●●●	●●	●●●
Craig-Schapiro et al.	●●●●	●●	●●●
Hilbrands et al.	●●●●	●●	●●●
Mamode et al.	●●●●	●●	●●
Willicombe et al.	●●●●	●●	●●
Oto et al.	●●●●	●●	●●●
Kute et al.	●●●●	●●	●●●
Villanego et al.	●●●●	●●	●●●
Alshaqaq et al.	●●●●	●●	●●
Requiao-Moura et al.	●●●●	●●	●●

Supplementary Figure S1: Forest plot of patient characteristics that were significantly associated with mortality.

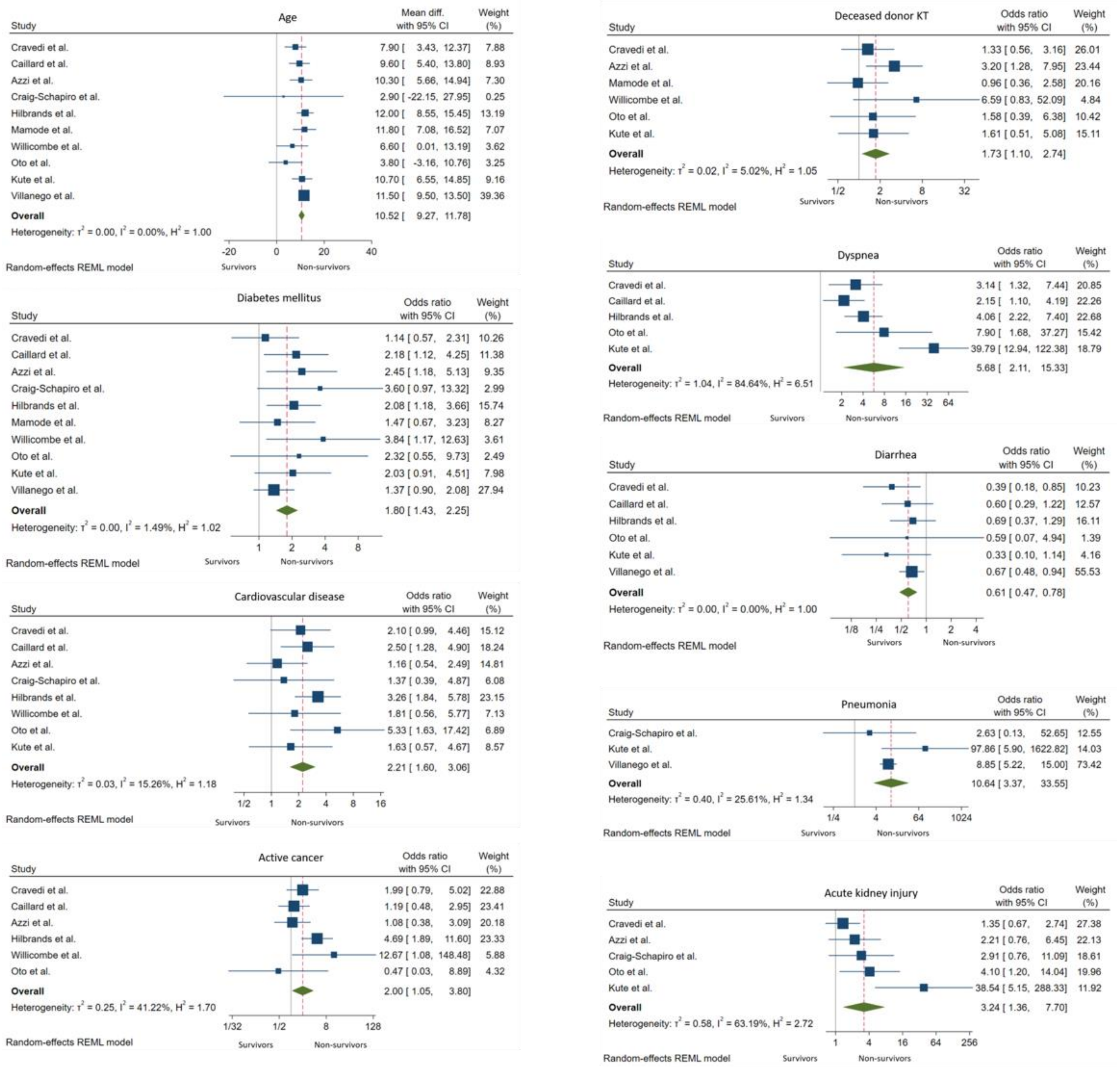

Supplementary Figure S2: Forest plot of laboratory investigations that were significantly associated with mortality.

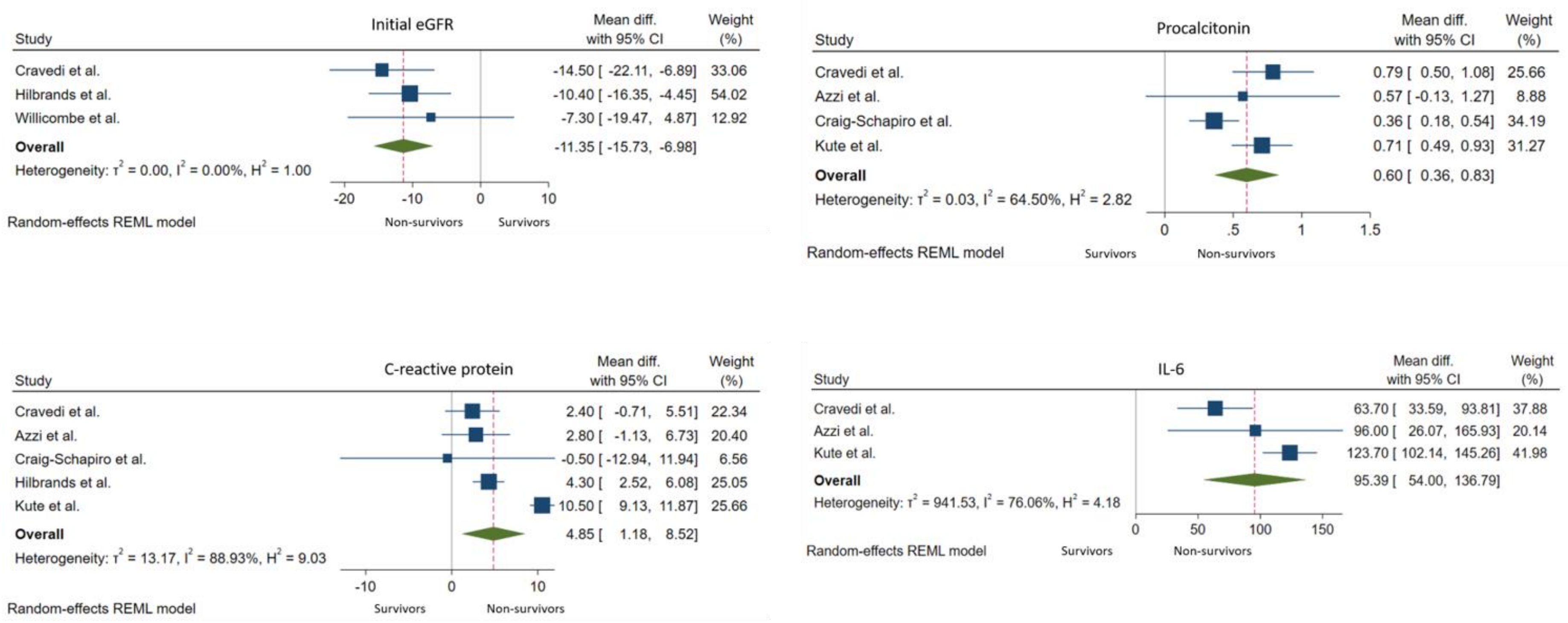

Supplementary Figure S3: Forest plot of treatments received that were significantly associated with mortality.

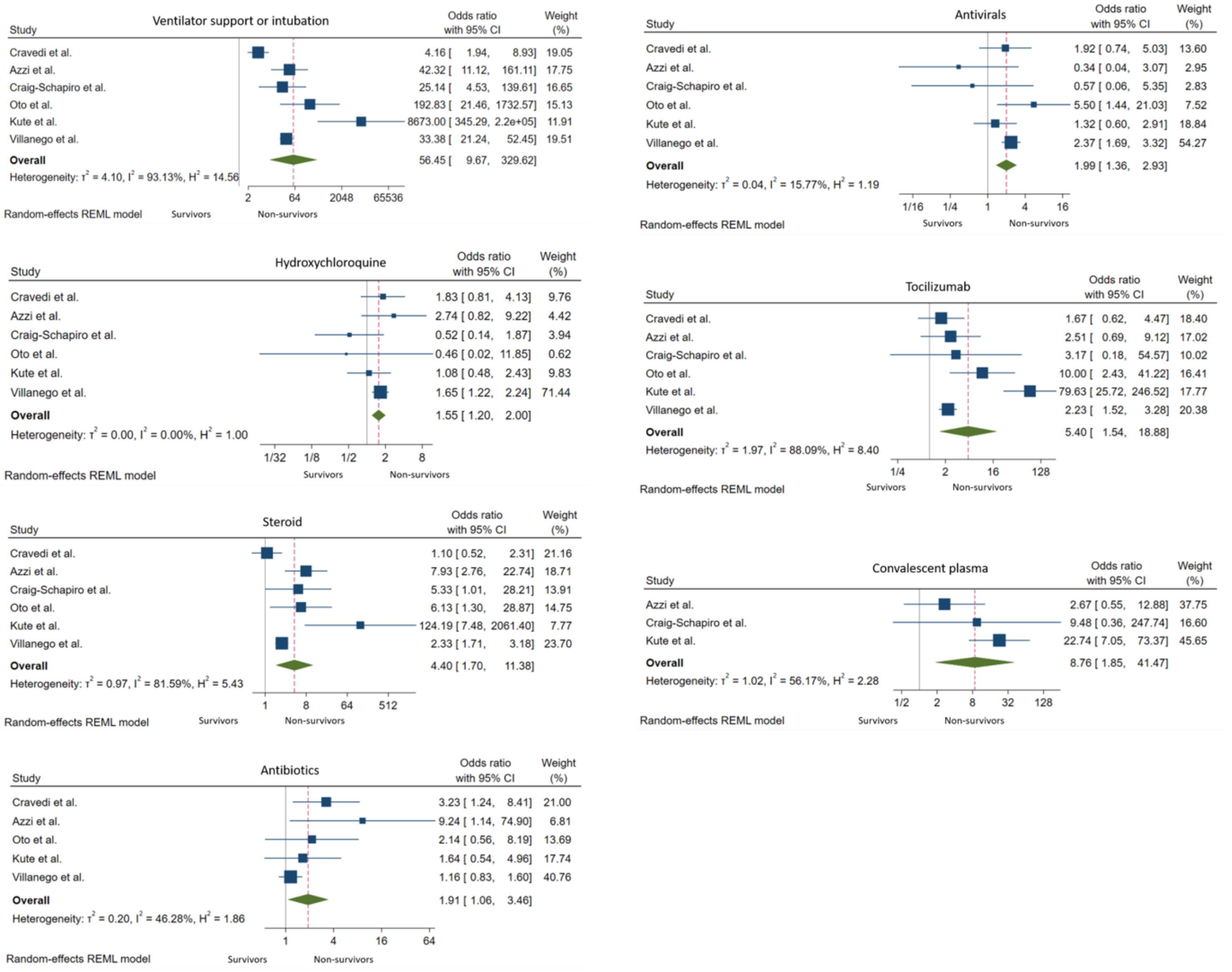

Supplement: Supplementary file 1 — Supplementary Information. [file 41598_2021_99713_MOESM1_ESM.pdf]
